# Measurement of inferior vena cava and aorta with bedside ultrasound to assess degree of dehydration in children

**DOI:** 10.1186/2036-7902-7-S1-A23

**Published:** 2015-03-09

**Authors:** Hyuksool Kwon, Jin Hee Lee, Kyuseok Kim, Young Ho Kwak, Do Kyun Kim

**Affiliations:** 1 Department of Emergency Medicine, Seoul National University Bundang Hospital, Bundang-gu, Seongnam-si, Gyeonggi-do Republic of Korea; 2 Department of Emergency Medicine, Seoul National University College of Medicine, Seoul, Republic of Korea

**Keywords:** Care Provider, Health Care Provider, Interventional Radiology, Teaching Hospital, Linear Regression Model

## Background

Clinical dehydration scale (CDS) has been developed and undergone validation for measuring dehydration in children. However, these included few objective subjects with dehydration requiring fluid replacement (DRFR). Moreover, interobserver reliability can be diverse and this can cause confusion between health care providers.

## Objective

This study was designed to validate the use of ultrrasound in the prospective identification of children with dehydration by investigating whether the inferior vena cava-aorta ratio (IVC/Ao) correlated with the CDS in children.

## Patients and methods

A prospective observational pilot study was carried out in a pediatric emergency department (PED) between October 2013 and June 2014 at the tertiary university teaching hospital in South Korea. Single investigator obtained transverse images using bedside US. The CDS was measured before the IVC/Ao calcualtion. Subjects were asked to return after improvement in the CDS and symptoms. Greater than or equal to 1 in the CDS was judged to be dehydration requiring fluid replacement (DRFR). Relation between the CDS, the DRFR, and the IVC/Ao was measured.

## Results

34 children older than 3 months were enrolled. There was correlation between the CDS and the IVC/Ao (Spearman's rho was = -0.76; 95% confidence interval [CI] = -0.87, -0.57). The linear regression model between the DRFR and the IVC/Ao resulted in an R2 value of 0.54 (p < 0.001) and a slope of -1.56 (95% CI = -2.06 to -1.06). The IVC/Ao discriminative ability of DRFR was assessed with an area under the receiver operating characteristic curve of 0.93 (95% = CI 0.84, 1.00). An IVC/Ao cutoff of 0.63 produced a sensitivity of 82% and a specificity of 100% for DRFR.

## Conclusion

IVC/Ao, as measured by bedside US, was an accurate measurement of dehydration in children.
